# Fatty acid analysis in microalgal mono- and polycultures using diffuse reflectance infrared Fourier transform spectroscopy coupled with partial least squares analysis

**DOI:** 10.1016/j.heliyon.2024.e33058

**Published:** 2024-06-14

**Authors:** Calle Niemi, Francesco G. Gentili

**Affiliations:** Department of Forest Biomaterials and Technology, Swedish University of Agricultural Sciences, 901 83, Umeå, Sweden

**Keywords:** Diffuse reflectance infrared fourier transform spectroscopy (DRIFT), Fatty acids, Microalgae, Partial least squares regression (PLSR)

## Abstract

Fatty acids are of particular interest for industrial applications of microalgal feedstock, as these have a wide array of different uses such as pharmaceuticals and biofuels. Fourier transform infrared (FTIR) spectroscopic techniques used in combination with multivariate prediction modeling are showing great potential as analytical methods for characterizing microalgal biomass. The present study investigated the use of diffuse reflectance Fourier transform infrared spectroscopy (DRIFTS) coupled with partial least squares regression (PLSR) to estimate fatty acid contents in microalgae. A prediction model for microalgal samples was developed using algae cultivated in both Bold's basal medium (BBM) and sterilized municipal wastewater under axenic conditions, as well as algal polycultures cultivated in open raceway ponds using untreated municipal wastewater influent. This universal prediction model was able to accurately predict microalgal samples of either type with high accuracy (RMSEP = 1.38, relative error = 0.14) and reliability (R2 > 0.92). DRIFTS in combination with PLSR is a rapid method for determining fatty acid contents in a wide variety of different microalgal samples with high accuracy. The use of spectral characterization techniques offers a reliable and environmentally friendly alternative to traditional labor intensive techniques based on the use of toxic chemicals.

## Highlights

• Multivariate FTIR analysis can accurately predict microalgal fatty acid contents.
• The method works regardless of culture medium and microalgal species.
• The same model can be used to predict both monocultures and complex polycultures.
• The study develops an environmental sustainable method for the microalgae industry.

## 1 Introduction

There is a growing demand for sustainable sources of lipids for the production of biofuels as well as nutritional additives and pharmaceuticals [[1], [2], [3]]. Microalgae have been subject to extensive research in the past several decades due to their ability to amass large quantities of lipids [4]. Microalgal cultures proliferate rapidly, accumulating biomass faster than most land crops, and do not require arable land, meaning that they also do not compete with existing food crops [5]. They are also a versatile industrial feedstock in the sense that they can be cultivated in sealed bioreactors when a particularly high level of purity of the resulting extract is needed, while also having the option of growing in large open ponds when the purity of the feedstock is less important than the quantity. Some studies have suggested cultivating microalgae in wastewater as a form of water remediation [6], while using the resulting algal feedstock for biodiesel production [7] or as plant biostimulant [8].

The fatty acid content of microalgae is highly variable, and depends on a large variety of factors such as the growth phase and the specific culture conditions [9]. Light availability can also be tuned to increase fatty acid yields, as photooxidative stress has been shown to cause carbon reallocation to storage triacylglycerides (TAGs) [10,11]. The relative fatty acid abundance can also be increased by alteration of salt concentration, potentially doubling the quantity compared to regular conditions [[12], [13], [14], [15]]. One of the most commonly investigated methods for increasing fatty acid yield is nitrogen starvation [16], which can produce fatty acid contents that are three or even five times that of regular growth conditions, depending on species and culture duration [17,18].

Some oleaginous algae have been shown to produce as much as 80 % of their dry weight (DW) in oil [2], albeit this oil typically contains not only fatty acids but also other lipid-soluble substances like carotenoids [19], phytosterols [20] and hydrocarbons- [21]. , Fatty acid contents of 13–31 %DW are often achievable under regular conditions, with nutrient stress typically achieving an average of 41 %DW [22].

A rapid and accurate method for estimating the fatty acid contents of microalgal biomass would be highly beneficial when screening for strains with optimal fatty acid accumulating properties, as well as when evaluating the correct time for harvesting a large microalgal culture. Spectroscopic techniques have been shown to be highly accurate in quantifying common components of algal biomass with minimal sample processing and labor requirements, and these methods have the potential to replace traditional methods for the characterization of microalgae which are laborious and require extensive use of chemicals [23,24]. The development of spectroscopic tools and techniques for fatty acid analysis in microalgae should therefore enable researchers to more rapidly identify the best strains and culture conditions for maximizing fatty acid yield, as well as improve monitoring of industrial-scale algal cultures. Fourier transform infrared spectroscopy (FTIR) has been used to estimate the shift in carbon partitioning of *Chlamydomonas reinhardtii* and *Scenedesmus subspicatus* during nitrogen-starvation over time, by correlating the ratio of intensity of specific absorbance bands with lipid, carbohydrate and protein contents, with decent accuracy [25]. Considerably higher accuracy of prediction of total fatty acid contents was obtained in *Nannochloropsis oculata* through the use of FTIR coupled with partial least squares regression (PLSR) [26]. Attenuated total reflectance (ATR) FTIR coupled with PLSR has also been used to determine fatty acid contents and even the quantities of certain fatty acid species in *N. oceanica* cells [27]. Other spectral PLSR analyses of fatty acids in microalgae have relied on visible or near-infrared (NIR) [28,29] or H-1 nuclear magnetic resonance (NMR) spectroscopies [30]. The use of FTIR and PLSR in the analysis of microalgal fatty acid contents is thus largely limited to one genus of green algae, and a broadening of these methods to encompass a wider array of microalgal samples is necessary. Diffuse reflectance Fourier transform infrared spectroscopy (DRIFTS) is a particularly interesting technique due to its high resolution, and the linearity of spectral absorbance intensities across the spectrum which tends to lead to improved predictive accuracy when compared to ATR which suffers from diminished absorbance at higher wavenumbers [31,32]. To the authors’ knowledge, there are presently no studies showing the use of DRIFTS in prediction of microalgal fatty acid contents however.

In the present study, DRIFTS was used to estimate the fatty acid contents in dehydrated microalgal biomass in combination with PLSR analysis. The feasibility of using these techniques for pure monocultures cultivated in controlled lab conditions was compared to that of open raceway ponds where numerous microalgal species can be expected to be present, in addition to other microbial organisms. Quantifying the fatty acid contents of microalgae is necessary for determining the potential for using the biomass for biofuel production or other uses like development pharmaceuticals or nutraceuticals, and a rapid method for estimating microalgal fatty acid contents would be greatly beneficial.

## 2 Materials and methods

### 2.1 Microalgal cultures and harvest

The microalgae analyzed in the present study were grouped into two separate data sets: one consisting of monocultures of green microalgae, cultivated in either synthetic growth media or in filtered wastewater and one set consisting of polycultures grown in open raceway ponds in untreated municipal wastewater influent. For multivariate spectral analysis, the two data sets were analyzed both separately and as one large, combined data set.

The monoculture set consisted of a variety of species cultivated in either Bold's basal medium (BBM) [33] or lightly filtered and autoclaved municipal wastewater at either 22 °C or 5 °C with a light:dark cycle of 16:8 h. These cultures were grown for varied durations, from 6 to 46 days, meaning that some were in the late exponential growth phase while some were in the stationary phase and had likely entered into nutrient stress. This ensured a data set with a wide variety of fatty acid quantities, making it suitable for the calibration of multivariate prediction models. Strains used included *Chlorella vulgaris* ‘13–1’, *Scenedesmus obliquus* ‘B2-2’, and *Coelastrella* spp. ‘3–4’, with some additional samples of *Haematococcus* spp., *Coelastrum* spp. ‘RW-10’.

The polycultures consisted of samples harvested throughout two growth seasons at the Dåva algae pilot plant in Umeå, Sweden, cultivated in untreated municipal wastewater influent throughout the summer seasons of 2017 and 2019. The 2017 season samples have been previously characterized by Lage and Gentili [34], and the microalgal diversity has been well documented. The 2019 season samples were characterized during the present study.

Microalgal cultures were concentrated by centrifugation at 4000 x G for 10 min. Liquid media was removed and the concentrated cultures were frozen at −20 °C overnight before lyophilization. Frozen microalgae were lyophilized in a freeze dryer (Edwards high vacuum international, Crawley, England) at −40 °C overnight.

### 2.2 Cell disruption

To ensure high extraction efficiency, cell were disrupted following the protocol from Ref. [8]. Briefly, microalgal cells were flash frozen using liquid N_2_, and grinded using 2.4 mm steel beads at 20 Hz for 2 min in a Mixer Mill MM 400 (Retsch GmbH, Haan, Germany). The samples were placed on ice to cool down, in order to prevent degradation of heat-sensitive fatty acids, and the grinding cycle was repeated for a total of twenty cycles. Light microscopy was used to assess the disruption of cells and cell clusters which could inhibit fatty acid extraction.

### 2.3 Fatty acid extraction and purification

#### 2.3.1 Lipid extraction

Extraction of algal lipids was performed using a protocol described by Ref. [35], with minor alterations. A 2:1 solvent mixture of chloroform:methanol (MeOH) was used to extract soluble compounds from 20 ± 0.2 mg algal sample. An 0.73 % (w/v) aqueous NaCl solution was added to reach a chloroform:MeOH:NaCl ratio of 2:1:0.8 (v/v/v) followed by repeated inversion of the sample. The sample was centrifuged at 400 x G for 2 min, and the lower, organic phase was recovered. The aqueous phase was washed twice with chloroform and all organic phases were pooled. To the pooled lipid sample, 10 μg of tripentadecanoic acid (C15:0-TAG) (product number: 33–1500, Larodan, Solna, Sweden) was added as internal standard for quantification. The solvent was evaporated from the crude lipid extract overnight using a Syncore Multievaporator (Büchi Labortechnik GmbH, Essen, Germany) at 45 °C with a pressure of 270 mbar and orbital shaking at 200 rpm.

#### 2.3.2 Solid phase extraction

The lipid extract was purified using solid phase extraction (SPE), as previously described [36]. SPE cartridges (Thermo Fisher) were primed with hexane prior to sample purification, and fixed to a vacuum manifold. The crude lipid extracts were dissolved in hexane, washing the tube twice to ensure maximum recovery, and placed in the SPE cartridge. Neutral lipids were eluted using a mixture of 80:20:10 hexane:diethyl ether:acetic acid (v/v/v), and polar lipids were eluted using a mixture of 2:2:1 MeOH:acetone:hexane. However, the purified lipid fractions were pooled in the same sample vial and the solvents were evaporated overnight as described above.

#### 2.3.3 Transmethylation

The purified lipid extract was dissolved in toluene, and 1 % H_2_SO_4_ in dry MeOH was added as transmethylation reagent. The sample vial was flushed with N_2_ in order to limit oxidation and transmethylation was performed at 80 °C for 2 h with orbital shaking at 100 rpm. After cooling to room temperature, an equal quantity of aqueous 5 % NaCl and hexane was added, the sample was vortexed and centrifuged for 2 min at 400×*g* to obtain clear phase separation. The organic phase was recovered, the aqueous phase was washed again with hexane and the two organic phases were pooled. Aqueous 2 % KHCO3 was used as washing agent of the organic phase that was dried by the addition of a small quantity of anhydrous Na_2_SO_4_ to remove trace amounts of water. The organic phase was recovered and evaporated overnight as described above. Since toluene evaporates more slowly than the other solvents used, residual solvents were evaporated at 55 °C with a pressure of 120 mbar for 1–2 h until all solvent was removed. The fatty acid methyl ester (FAME) sample was dissolved in heptane and analyzed using gas chromatography flame ionization detection (GC-FID).

### 2.4 Gas chromatography analysis

The FAME samples were analyzed by gas chromatography flame ionization detection (GC-FID). A TriPlus RSH autosampler (Thermo Scientific, Hägersten, Sweden) was used to inject 1 μL sample in 1:10 split mode into a Trace 1310 gas chromatograph (Thermo Scientific). The chromatograph was equipped with a 30 m × 0.32 mm ID FAMEWAX capillary column (Restek, Bellefonte, Pennsylvania, USA), with a 0.25 μm Crossbond polyethylene glycol stationary phase. The column temperature was held at 195 °C at the time of injection, and was increased at a rate of 1.8 °C min^−1^ up to 240 °C, where it was held for 2.8 min prior to cool-down. Data acquisition and peak integration was performed using Chromeleon 7 software (Thermo Scientific). Peaks were identified using a marine FAME standard mixture (Product number: 35066, Restek).

FAME quantification was performed using a method described by Ref. [37]. Calibration curves were made for all individual fatty acids in the standard mixture, and their respective relative response factors (RRFs) were calculated by normalising to the pentadecaenoic methyl ester internal standard.

All the above mentioned studies were performed at the department of Forest Biomaterials and Technology, Swedish University of Agricultural Sciences in Umeå Sweden.

### 2.5 Diffuse reflectance Fourier transform infrared spectroscopy (DRIFTS)

DRIFTS measurements were performed on the microalgal samples, using a protocol described in Ref. [38]. Briefly, dried algal powders were mixed with potassium bromide (KBr) to an approximate 1:10 ratio of algae:KBr (v:v). Spectra were measured using an IFS 66 v/S vacuum spectrometer (Bruker Optik GmbH, Ettlingen, Germany), co-adding 128 scans over a spectral region of 400–4000 cm^−1^, at a resolution of 4 cm^−1^. The KBr background was subtracted by OPUS v.5 software (Bruker Optik GmbH), and further processed using the MCR-ALS GUI, available at the Vibrational Spectroscopy Core Facility, Department of Chemistry, Umeå University (v4c, https://www.umu.se/en/research/infrastructure/visp/downloads/) running in MATLAB software (version R2017b, MathWorks, Natick, MA, USA).

The 800-1800 cm^−1^ fingerprint region is known to contain bands with a high degree of correlation to fatty acids as well as carbohydrates and proteins, the main components of algal biomass, and the spectra were thus cut to this region. Asymmetric least squares (AsLS) baselining was performed on the cut spectra (lambda = 20 000, p = 0.001), followed by normalization to the total spectral area. The processed spectra were used for multivariate analysis. There are spectral signatures relevant to fatty acids in the higher wavenumbers (especially in the 2800-3000 cm^−1^ range) (Rohman & Man, 2010), and so an attempt was made at using a broader spectrum (500-3500 cm^−1^) for multivariate modeling, but PLSR was unable to resolve spectral components without excessive noise, and the prediction was considerably lower than with the narrower spectral range (data not shown).

### 2.6 Statistical analysis

Any statistically significant differences in total fatty acid contents between different algal culture conditions were tested using *t*-test. In the case of variables such as the month of harvest of polycultures or culture duration of monocultures where more than two groups had to be compared, one-way analysis of variance (ANOVA) was performed. For comparison of monoculture culture duration, samples were grouped approximately into weeks rather than days, for the sake of simpler data analysis. Statistical analysis was performed in Excel (16.16.27, Microsoft, Redmond, Washington, US).

### 2.7 Multivariate spectral analysis

The sample sets were analyzed using principal component analysis (PCA), to determine the influence of sample characteristics like species, growth temperature, culture medium and culture duration on the DRIFTS spectra. Such data were not available for the polyculture samples, and these were instead analyzed in terms of harvest month. Due to the differences between the monoculture and polyculture data sets in terms of available data, PCA was performed separately on both data sets. PCA was performed in the RStudio software (Rstudio, Boston, MA, USA) using the FactoMineR package and visualized using factoextra.

Partial least squares regression (PLSR) was also used to construct prediction models, using a method described previously [39]. PLSR analysis was done using RStudio with scripts from the PLS package. The two data sets were analyzed both separately and together (total n = 101), and for all three runs approx. 75 % of the samples were used for calibration and 25 % for model validation. The component number was chosen through leave-one-out cross-validation, and the predictive accuracy of the resulting models was determined through the root mean square error of prediction (RMSEP), the relative error of prediction (RE), and the R^2^ coefficient of determination of the validation samples.

## 3 Results and discussion

### 3.1 Fatty acid contents

The total fatty acid contents of the microalgal monocultures differed considerably from the polycultures. The monocultures featured a far wider range of fatty contents, from 1.32 to 22.46 %DW, while the polyculture fatty acid contents were universally quite low in comparison, ranging from 0.76 to 4.21 %DW. This can be explained by the semi-continuous mode of operation of the ponds, being repeatedly harvested and having the growth medium replenished regularly, likely leading to a culture that is constantly in the early to mid-exponential growth phase. This also explains the low amount of fatty acids, as fatty acids are accumulated in stress situations as long-term energy storage. The monoculture samples on the other hand were harvested in various stages of the growth cycle, leading to a wider range of total fatty acid contents.

There were some differences between monoculture species, with *Coelastrella* sp. exhibiting the highest (10.43 ± 5.84 %DW), followed by *Chlorella vulgaris* (9.85 ± 2.66 %DW), *Haematococcus* sp. (8.50 ± 2.50 %DW), and *Scenedesmus obliquus* (7.83 ± 4.46 %DW). Two-sample t-tests were used to determine statistically significant differences in total fatty acid quantities depending on culture conditions. A large difference was found between culture temperatures, with fatty acid contents in cultures grown at 5° on average being close to three times higher (15.06 ± 2.21 %DW) than corresponding cultures grown at 22° (6.38 ± 1.40 %DW), t(8) = 8.106, p < 0.01, an effect that has been described in these strains by Lindberg et al. [40]. The total fatty acid contents did not change to a statistically significant extent depending on culture medium where all other culture conditions remained the same, with *Chlorella vulgaris* showing minor differences (WW = 8.77 ± 0.02, BBM = 10.53 ± 1.43 %DW), t(2) = 2.139, p = 0.166, and *Scenedesmus obliquus* remaining largely unchanged (WW = 6.20 ± 0.40, BBM = 6.69 ± 0.16 %DW), t(2) = 1.964, p = 0.144. The *Coelastrella* sp. strain on the other hand appeared considerably more sensitive to the culture medium (WW = 17.68 ± 4.39, BBM = 10.03 ± 0.76 %DW), while still not showing a statistically significant difference, t(2) = 2.973, p = 0.097. One-way ANOVA also showed statistically significant differences depending on culture duration (F(5, 37) = 4.365, p = 0.003). This is to be expected as the cultures were in different growth cycle stages, with the short-term cultures being in the early exponential phase and thus having lower fatty acid contents, with the long-term cultures having entered into the static phase and having exhausted nutrients in the growth medium, triggering lipid accumulation [22].

The polyculture samples did not differ significantly in total fatty acid contents depending on the year of culture, with the 2019 season being only slightly higher (1.96 ± 0.74 %DW) than the 2017 season (1.88 ± 0.67 %DW) on average, t(55) = 0.426, p = 0.672. Monthly differences in fatty acid contents were statistically significant according to a one-way ANOVA (F(3, 53) = 2.907, p = 0.043), likely as a consequence of temperature differences.

### 3.2 FTIR spectroscopy

The FTIR spectrum contains spectral absorbance bands which are highly characteristic of certain biological molecules and can often be directly associated with wider categories of compounds, e.g. fatty acids. From looking at the FTIR spectrum, one can therefore draw general conclusions about the ratio of the major compounds in a biomass sample, especially since these predominantly consist of macromolecules like polysaccharides or proteins, or fatty acids which can be distinguished by a number of spectral bands, especially in the so-called fingerprint region [41].

**Fig. 1:**
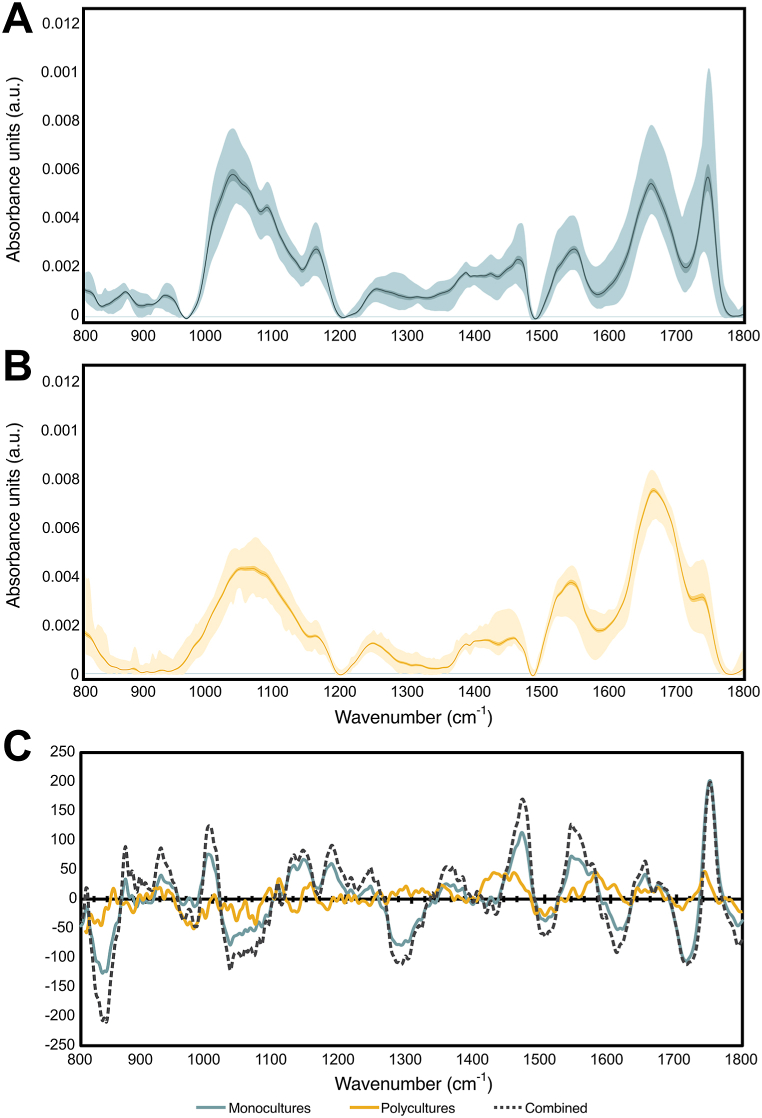
DRIFTS spectral profiles of microalgal monocultures (A), and polycultures (B). The light-shaded area indicates the minimum and maximum absorbance for all samples within the data set, the dark-shaded area indicates the 95 % CI, and the black line is the mean spectrum. The PLSR regression coefficients (C) of the monocultures, polycultures and combined data sets.

The spectral variance of the polyculture data set was also considerably narrower than the monocultures (Fig. 1A and B). From the spectra, it appears that the polycultures contain far lower fatty acid contents, and a large amount of protein, judging by the intensity of the Amide I and II peaks at approx. 1540 and 1660 cm^−1^, respectively. This has been confirmed by amino acid analysis, showing that the protein contents of this data set are above 20 %DW in the majority of the samples, and all but one being above 16 %DW (data not shown). The spectral intensity of the polyculture samples was considerably lower as well, likely due to high ash contents (in the range of 10–30 %DW), caused by being cultured in untreated municipal wastewater in open ponds.

### 3.3 Multivariate spectral analysis

#### 3.3.1 Principal component analysis (PCA)

PCA was performed to determine to which extent the DRIFTS spectra of the microalgae depended on the species, growth temperature, culture medium or culture duration of the samples.

**Fig. 2:**
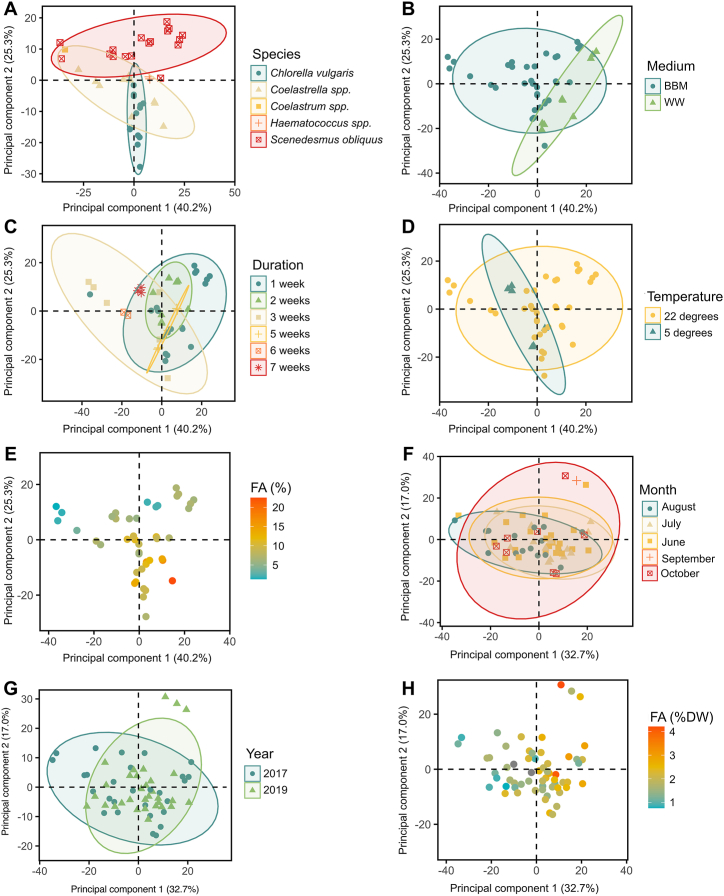
PCA scatterplots for the monocultures (A–E) and polycultures (F–H) datasets. The ellipses show the 90 % confidence interval of the indicated category.

For the monocultures, PCA could resolve the data into four principal components (PCs) explaining a total of 85.65 % of variance. PC1 accounted for 40.2 % of the variance, while PC2 accounted for 25.3 %. There was notable clustering dependent on species, with *Scenedesmus obliquus* samples grouping separately from the others, while *Chlorella vulgaris* and *Coelastrella* spp. tended to overlap considerably (Fig. 1A). Most variance in the *C. vulgaris* samples was also along PC2, while the others depended more on PC1. The other species had too few samples to say anything conclusive about them. Cultures from both types of growth media overlapped extensively, but the wastewater cultures tended to reside in the right-hand quadrants, implying a higher correlation to PC1 (Fig. 2B). The samples did not notably cluster according to temperature or culture duration, likely due to different species reacting differently to these culture conditions (Fig. 2C and D).

**Fig. 3:**
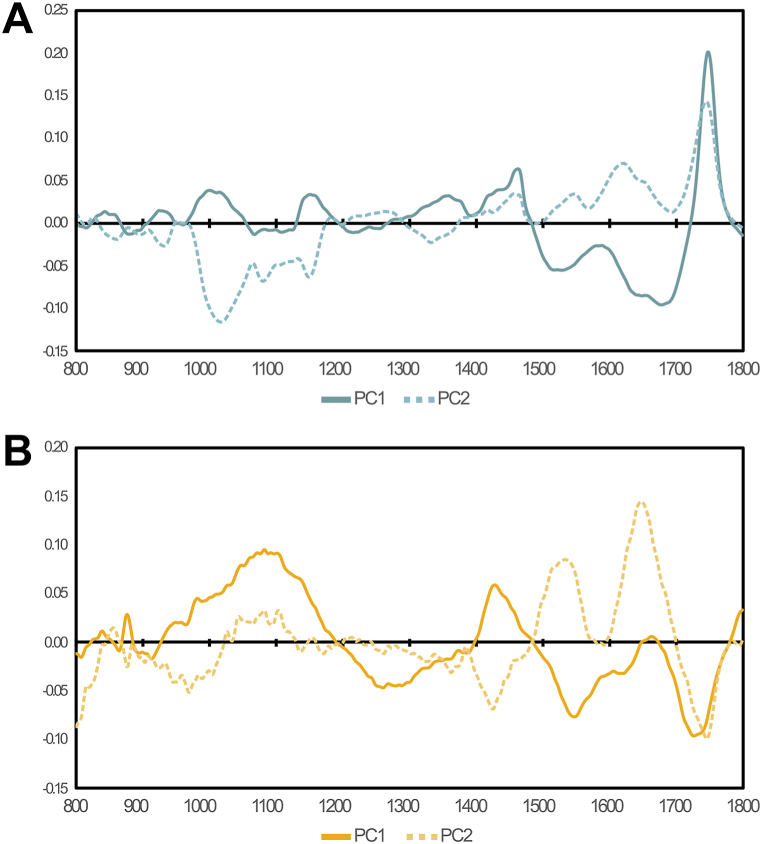
PCA spectral loadings of microalgal monocultures (A) and polycultures (B). The first two principal components are displayed.

The distribution of the monoculture samples seemed relatively dependent on fatty acid contents, with higher-quantity samples almost universally grouping in the lower right quadrant and low-quantity samples being in either of the upper quadrants, showing an apparent correlation of fatty acid contents to PC1, and to a lesser extent PC2 (Fig. 2E). This was confirmed by the PCA loadings for PC1 showing major influence from the fatty acid ester peak at 1740 cm^−1^ (Fig. 3A). PC2 also heavily correlated to the 1740 cm^−1^ peak, but had negative contribution from the C–O–C stretches in the 950-1200 cm^−1^ region associated with carbohydrates [42].

The fit of the polyculture model was considerably lower than that of the monoculture model, requiring 6 components to explain 84.07 % of the variance in the data. Plotting PC1 (32.7 %) and PC2 (17.0 %) against each other, the spectra of the polyculture samples showed no separation based on the month or year of harvest (Fig. 2F and G), and the distribution of samples according to fatty acid contents seemed largely random (Fig. 2H). The explanatory power of this PCA model was thus quite low. This can likely be explained by the high degree of similarity between the polyculture samples, having quite a homogenous composition regardless of harvest time. The polyculture set simply did not have enough compositional variance to calibrate a reliable model. PCA loadings for the polyculture PC1 indicated no correlation to the relevant fatty acid absorbance bands (Fig. 3B), unlike the monoculture model.

#### 3.3.2 Fatty acid prediction by PLSR

PLSR was used to predict fatty acid contents in microalgal samples. The monoculture and polyculture data sets were evaluated separately and in a combined prediction model. The monoculture PLSR model performed well, with an RMSEP of approx. 1.2615, an RE of 0.0231, and R^2^ > 0.93 (Table 1). Considering the fatty acid contents in the validation set ranged from 1.91 to 15.23 %DW, this predictive error is quite low, and the model can be considered reliable. The model regression coefficient also indicated that the model relied on relevant spectral bands associated with fatty acids (Fig. 1C). In particular, the notable ester carbonyl peak at 1750 cm^−1^, but also smaller bands at 1470 and 990 cm^−1^, likely associated with CH_2_ and CH_3_ bending vibrations and CH bending, respectively [41].

**Table 1:** PLSR prediction results.

Culture type	Component number	RMSEP	RE	R^2^
Monocultures	5	1.26	0.02	0.9399
Polycultures	5	0.54	0.25	0.0461
Combined	6	1.38	0.14	0.9212

The polyculture model was unable to predict fatty acid contents in the validation set. Even with only two components, the model incorporated considerable amounts of noise. As with the monoculture set, five components was calculated to be optimal, but the resulting predictions did not correlate with the expected values at an R^2^ < 0.05 (Table 1). The regression coefficient for the five-component model also showed no correlation to expected spectral bands (Fig. 1C). As with the PCA, it was clear that the narrow range of calibration made the model unreliable.

**Fig. 4:**
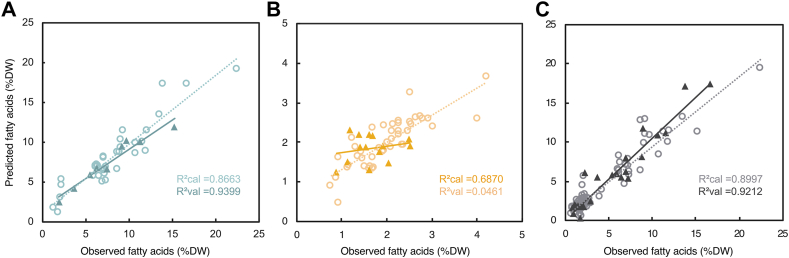
Calibration and validation results from PLSR, for the monocultures model (A), the polycultures model (B), and the combined model (C). R2cal indicates the coefficient of determination of the calibration samples, and R2val indicates the coefficient of determination of the validation samples. Calibration points are indicated by open circles, and validation points by black triangles. The dotted grey line shows the trendline of predicted vs observed calibration points, and the solid black line indicates the trendline of predicted vs observed validation points.

When combining both data sets into one model, however, prediction performance was almost on-par with the monoculture model, with an RMSEP of approx. 1.3772, an RE of 0.1369, and R^2^ > 0.92 (Table 1). The predictive error was higher than the monoculture model, but still within an acceptable range. While the polyculture set on its own was not capable of producing a viable prediction model, the polyculture spectra were sufficiently similar to those of the monocultures to be predictable when combined into one large set. The cross-validation fits and prediction results of each PLSR model are shown in Fig. 4A–C.

For the sake of comparison to univariate methods of spectroscopic quantification, the peak heights of the fatty acid ester C=O stretching vibration at 1740 cm^−1^ and the protein-associated Amide I band at 1650 cm^−1^ [43] were measured, and the fatty acid/Amide I peak ratio was calculated. The fatty acid peak itself was linearly correlated to fatty acid contents, with an R^2^ > 0.82. When taking the ratio of the fatty acid and Amide I peaks, this correlation improved slightly to R^2^ > 0.83. As has been shown by previous studies [25], these peaks can be used to estimate fatty acid contents in microalgae on their own, but the multivariate PLSR method outperforms in terms of accuracy at the cost of some minor computation time. This study shows the efficacy and accuracy of DRIFTS used in combination with PLSR for the determination of microalgal fatty acids, regardless of species or conditions of growth, presenting an improvement over univariate methods. This is consistent with the findings of Zhang et al. [27] who showed that individual absorbance bands perform considerably worse than whole spectra for fatty acid quantification in *Nannochloropsis oceanica*. The results of the present study show the viability of whole-spectra analysis of multiple microalgal species using the same prediction model.

The lack of variety in the polyculture samples in the present study means that further studies are needed to verify that the method works in complex polycultures with high fatty acid contents as well, however. Future research should be aimed at using a systematically constructed data set of microalgal cultures with a variety of species compositions, grown in conditions known to induce extreme fatty acid contents, such as light, salt, or nitrogen-stress [10,15,17].

## 4 Conclusions

Estimation of fatty acid contents in microalgae using DRIFTS was shown to be highly effective and accurate, given robust model calibration. The study found minimal effect on spectral profiles depending on culture medium, growth phase, temperature, or species, and the method works on complex samples like polycultures grown in wastewater. A universal prediction model trained on a sufficiently broad data set should be able to estimate fatty acids in microalgae grown in a diverse set of conditions. There is high potential for the applicability of multivariate spectral prediction techniques in algal industries, since these methods require far less labor time and chemical use. The use of spectral characterization techniques in algal research and industries has the potential to cut down on the use of chemicals while also providing results faster than traditional chemical methods. In particular, this should enable easier screening of microalgal strains and culture conditions for optimization of lipid production for the development biofuels.

## Data availability statement

The data will be made available upon request.

## CRediT authorship contribution statement

**Calle Niemi:** Writing – original draft, Methodology, Investigation, Formal analysis, Data curation. **Francesco G. Gentili:** Writing – review & editing, Supervision, Resources, Project administration, Funding acquisition, Conceptualization.

## Declaration of competing interest

The authors declare that they have no known competing financial interests or personal relationships that could have appeared to influence the work reported in this paper.

## References

[1] Ummalyma S.B., Sirohi R., Udayan A. (2023). Sustainable microalgal biomass production in food industry wastewater for low-cost biorefinery products: a review. Phytochemistry Rev..

[2] Chisti Y. (2007). Biodiesel from microalgae. Biotechnol. Adv..

[3] Olaizola M. (2003). Commercial development of microalgal biotechnology: from the test tube to the marketplace. Biomol. Eng..

[4] Mata T.M., Martins A.A., Caetano N.S. (2010). Microalgae for biodiesel production and other applications: a review. Renew. Sustain. Energy Rev..

[5] Chisti Y. (2008). Response to Reijnders: do biofuels from microalgae beat biofuels from terrestrial plants?. Trends Biotechnol..

[6] Gouveia L., Oliveira A.C. (2009). Microalgae as a raw material for biofuels production. J. Ind. Microbiol. Biotechnol..

[7] Shahid A., Malik S., Zhu H., Xu J., Nawaz M.Z., Nawaz S., Asraful Alam M., Mehmood M.A. (2020). Cultivating microalgae in wastewater for biomass production, pollutant removal, and atmospheric carbon mitigation; a review. Sci. Total Environ..

[8] Alling T., Funk C., Gentili F.G. (2023). Nordic microalgae produce biostimulant for the germination of tomato and barley seeds. Sci. Rep..

[9] Morales M., Aflalo C., Bernard O. (2021). Microalgal lipids: a review of lipids potential and quantification for 95 phytoplankton species. Biomass Bioenergy.

[10] Choi Y.K., Kumaran R.S., Jeon H.J., Song H.J., Yang Y.H., Lee S.H., Song K.G., Kim K.J., Singh V., Kim H.J. (2015). LED light stress induced biomass and fatty acid production in microalgal biosystem, Acutodesmus obliquus. Spectrochim. Acta Mol. Biomol. Spectrosc..

[11] Van Wagenen J., Miller T.W., Hobbs S., Hook P., Crowe B., Huesemann M. (2012). Effects of light and temperature on fatty acid production in Nannochloropsis salina. Energies.

[12] Ahn J.-W., Hwangbo K., Yin C.J., Lim J.-M., Choi H.-G., Park Y.-I., Jeong W.-J. (2014). Salinity-dependent changes in growth and fatty acid composition of new Arctic Chlamydomonas species, ArM0029A. Plant Cell Tissue Organ Cult..

[13] Hounslow E., Evans C.A., Pandhal J., Sydney T., Couto N., Pham T.K., Gilmour D.J., Wright P.C. (2021). Quantitative proteomic comparison of salt stress in Chlamydomonas reinhardtii and the snow alga Chlamydomonas nivalis reveals mechanisms for salt-triggered fatty acid accumulation via reallocation of carbon resources. Biotechnol. Biofuels.

[14] Kudahettige N.P., Pickova J., Gentili F.G. (2018). Stressing algae for biofuel production: biomass and biochemical composition of *Scenedesmus dimorphus* and *Selenastrum minutum* grown in municipal untreated wastewater. Front. Energy Res..

[15] Pandit P.R., Fulekar M.H., Karuna M.S.L. (2017). Effect of salinity stress on growth, lipid productivity, fatty acid composition, and biodiesel properties in Acutodesmus obliquus and Chlorella vulgaris. Environ. Sci. Pollut. Res. Int..

[16] Shen X.F., Chu F.F., Lam P.K., Zeng R.J. (2015). Biosynthesis of high yield fatty acids from Chlorella vulgaris NIES-227 under nitrogen starvation stress during heterotrophic cultivation. Water Res..

[17] Andeden E.E., Ozturk S., Aslim B. (2020). Effect of alkaline pH and nitrogen starvation on the triacylglycerol (TAG) content, growth, biochemical composition, and fatty acid profile of Auxenochlorella protothecoides KP7. J. Appl. Phycol..

[18] Zhu S., Wang Y., Shang C., Wang Z., Xu J., Yuan Z. (2015). Characterization of lipid and fatty acids composition of Chlorella zofingiensis in response to nitrogen starvation. J. Biosci. Bioeng..

[19] Sun X.M., Ren L.J., Zhao Q.Y., Ji X.J., Huang H. (2018). Microalgae for the production of lipid and carotenoids: a review with focus on stress regulation and adaptation. Biotechnol. Biofuels.

[20] Le Goff M., Le Ferrec E., Mayer C., Mimouni V., Lagadic-Gossmann D., Schoefs B., Ulmann L. (2019). Microalgal carotenoids and phytosterols regulate biochemical mechanisms involved in human health and disease prevention. Biochimie.

[21] Metzger P., Largeau C. (2005). Botryococcus braunii:: a rich source for hydrocarbons and related ether lipids. Appl. Microbiol. Biotechnol..

[22] Griffiths M.J., Harrison S.T.L. (2009). Lipid productivity as a key characteristic for choosing algal species for biodiesel production. J. Appl. Phycol..

[23] Murdock J.N., Wetzel D.L. (2009). FT-IR microspectroscopy enhances biological and ecological analysis of algae. Appl. Spectrosc. Rev..

[24] Porízka P., Prochazková P., Prochazka D., Sládková L., Novotny J., Petrilak M., Brada M., Samek O., Pilát Z., Zemánek P., Adam V., Kizek R., Novotny K., Kaiser J. (2014). Algal biomass analysis by laser-based analytical techniques-A review. Sensors.

[25] Dean A.P., Sigee D.C., Estrada B., Pittman J.K. (2010). Using FTIR spectroscopy for rapid determination of lipid accumulation in response to nitrogen limitation in freshwater microalgae. Bioresour. Technol..

[26] Coat R., Montalescot V., Leon E.S., Kucma D., Perrier C., Jubeau S., Thouand G., Legrand J., Pruvost J., Goncalves O. (2014). Unravelling the matrix effect of fresh sampled cells for in vivo unbiased FTIR determination of the absolute concentration of total lipid content of microalgae. Bioproc. Biosyst. Eng..

[27] Zhang D., Li Q., Yan C., Cong W. (2021). Determination of intracellular lipid and main fatty acids of Nannochloropsis oceanica by ATR-FTIR spectroscopy. J. Appl. Phycol..

[28] Challagulla V., Walsh K.B., Subedi P. (2016). Microalgal fatty acid composition: rapid assessment using near-infrared spectroscopy. J. Appl. Phycol..

[29] Chu B.Q., Chen K., Pan X.X., Wu Q.Y., Liu S.W., Gong J.Y., Li X.L. (2021). Visible/Short-wave near-infrared hyperspectral analysis of lipid concentration and fatty acid unsaturation of *Scenedesmus obliquus* in situ. Comput. Electron. Agric..

[30] Azizan A., Bustamam M.S.A., Maulidiani M., Shaari K., Ismail I.S., Nagao N., Abas F. (2018). Metabolite profiling of the microalgal diatom chaetoceros calcitrans and correlation with antioxidant and nitric oxide inhibitory activities via H NMR-based metabolomics. Mar. Drugs.

[31] Hahn A.Rosen, Kliem P., Ohlendorf Persson, Zolitschka B. (2011). Comparative study of infrared techniques for fast biogeochemical sediment analyses. G-cubed.

[32] Koçak A., Wyatt W., Comanescu M.A. (2021). Comparative study of ATR and DRIFT infrared spectroscopy techniques in the analysis of soil samples. Forensic Sci. Int..

[33] Nichols H.W., Bold H.C. (1965). Trichosarcina polymorpha Gen. et Sp. Nov. J. Phycol..

[34] Lage S., Gentili F.G. (2023). Chemical composition and species identification of microalgal biomass grown at pilot-scale with municipal wastewater and CO(2) from flue gases. Chemosphere.

[35] Axelsson M., Gentili F. (2014). A single-step method for rapid extraction of total lipids from green microalgae. PLoS One.

[36] Lage S., Gentili F.G. (2018). Quantification and characterisation of fatty acid methyl esters in microalgae: comparison of pretreatment and purification methods. Bioresour. Technol..

[37] Breuer G., Evers W.A.C., de Vree J.H., Kleinegris D.M.M., Martens D.E., Wijffels R.H., Lamers P.P. (2013). Analysis of fatty acid content and composition in microalgae. Jove-Journal of Visualized Experiments.

[38] Felten J., Hall H., Jaumot J., Tauler R., de Juan A., Gorzsas A. (2015). Vibrational spectroscopic image analysis of biological material using multivariate curve resolution-alternating least squares (MCR-ALS). Nat. Protoc..

[39] Niemi C., Mortensen A.M., Rautenberger R., Matsson S., Gorzsas A., Gentili F.G. (2023). Rapid and accurate determination of protein content in North Atlantic seaweed by NIR and FTIR spectroscopies. Food Chem..

[40] Lindberg A., Niemi C., Takahashi J., Sellstedt A., Gentili F.G. (2022). Cold stress stimulates algae to produce value-added compounds. Bioresour. Technol. Rep..

[41] Rohman A., Man Y.B.C. (2010). Fourier transform infrared (FTIR) spectroscopy for analysis of extra virgin olive oil adulterated with palm oil. Food Res. Int..

[42] Hong T., Yin J.Y., Nie S.P., Xie M.Y. (2021). Applications of infrared spectroscopy in polysaccharide structural analysis: progress, challenge and perspective. Food Chem. X.

[43] Schmitt J., Flemming H.-C. (1998). FTIR-spectroscopy in microbial and material analysis. Int. Biodeterior. Biodegrad..

